# Reconciling psychological and neuroscientific accounts of reduced motivation in aging

**DOI:** 10.1093/scan/nsab101

**Published:** 2021-08-27

**Authors:** Alexander Soutschek, Alexandra Bagaïni, Todd A Hare, Philippe N Tobler

**Affiliations:** Department of Psychology, Ludwig Maximilian University Munich, Munich 80802, Germany; Department of Psychology, University of Basel, Basel 4055, Switzerland; Zurich Center for Neuroeconomics, University of Zurich, Zurich 8006, Switzerland; Neuroscience Center Zurich, University of Zurich, Swiss Federal Institute of Technology Zurich, Zurich 8006, Switzerland; Zurich Center for Neuroeconomics, University of Zurich, Zurich 8006, Switzerland; Neuroscience Center Zurich, University of Zurich, Swiss Federal Institute of Technology Zurich, Zurich 8006, Switzerland

**Keywords:** mental effort, physical effort, decision-making, frontopolar cortex, transcranial direct current stimulation

## Abstract

Motivation is a hallmark of healthy aging, but the motivation to engage in effortful behavior diminishes with increasing age. Most neurobiological accounts of altered motivation in older adults assume that these deficits are caused by a gradual decline in brain tissue, while some psychological theories posit a switch from gain orientation to loss avoidance in motivational goals. Here, we contribute to reconcile the psychological and neural perspectives by providing evidence that the frontopolar cortex (FPC), a brain region involved in cost–benefit weighting, increasingly underpins effort avoidance rather than engagement with age. Using anodal transcranial direct current stimulation together with effort–reward trade-offs, we find that the FPC’s function in effort-based decisions remains focused on cost–benefit calculations but appears to switch from reward-seeking to cost avoidance with increasing age. This is further evidenced by the exploratory, independent analysis of structural brain changes, showing that the relationship between the density of the frontopolar neural tissue and the willingness to exert effort differs in young *vs* older adults. Our results inform aging-related models of decision-making by providing preliminary evidence that, in addition to cortical thinning, changes in goal orientation need to be considered in order to understand alterations in decision-making over the life span.

## Introduction

Reduced motivation to engage in demanding activities is a widespread phenomenon in aging, even in otherwise healthy individuals (Depping and Freund, 2011). Previous studies observed reduced willingness to engage in effortful behavior with increasing age (Hess and Ennis, 2012; Westbrook *et al.*, 2013). However, these deficits might be specific for self-regarding rather than for other-regarding benefits (Beadle *et al.*, 2015; Lockwood *et al.*, 2021), and, moreover, some studies reported no difference in effort aversion with increasing age (Seaman *et al.*, 2016, 2018). Diminished motivation has been related to frailty in older adults (Semprini *et al.*, 2012) and is a hallmark of several age-related conditions, including dementia and Alzheimer’s disease (Mortby *et al.*, 2011). Conversely, higher levels of motivation are indicative of mental and physical health as well as general well-being at older ages (Mortby *et al.*, 2011). Because motivation is important for healthy aging and because the population of older adults is expected to strongly increase in the future (United Nations, 2017), it is crucial to understand the brain mechanisms underlying age-related changes in motivation. Knowledge about the neurobiological basis of motivational deficits in older adults may foster the development of interventions for the prevention or rehabilitation of these deficits and the associated health problems.

Research on younger adults identified a core network of brain regions involved in motivating goal-directed behaviors (for reviews, see Studer and Knecht, 2016; Soutschek and Tobler, 2018; Le Heron *et al.*, 2018a). Within this network, the frontopolar cortex (FPC) plays a key role for the motivation to engage in effortful behavior (Soutschek *et al.*, 2018). Increasing FPC excitability with non-invasive brain stimulation enhanced the willingness to engage in rewarded mental and physical effort in younger adults, consistent with the hypothesized role of the FPC for weighting costs against benefits of competing action alternatives (Mansouri *et al.*, 2017) and for healthy motivation more generally (Jorge *et al.*, 2010; O’Callaghan *et al.*, 2014; Canu *et al.*, 2015). However, it is unknown whether changes in the FPC structure contribute to the increased effort aversion in older adults. Age-related impairments in cognition are often thought to result from structural brain changes (West, 1996; Li *et al.*, 2007; Dennis and Cabeza, 2012; Samanez-Larkin and Knutson, 2015; Lighthall, 2020). From this perspective, we expect the stronger effort aversion in older than in younger adults to be mediated by the age-related loss of the FPC gray matter volume (GMV). Note that this presupposes that the function of the FPC for motivating effort engagement remains constant over the life span but gradually declines in efficacy as a result of structural neural changes.

In addition to the assumption of gradual declines in structure and function in aging neuroscience, psychological theories posit that goals in decision-making change over the life span. In particular, motivational goals in older adults may switch from reward maximization to loss minimization (Depping and Freund, 2011; Hennecke and Freund, 2017). Under the assumption that resources for mental and physical effort are limited (Boksem and Tops, 2008), the exertion of effort can be considered as the loss of limited resources that needs to be weighed against the value of the reward at stake. With diminishing available resources for effort exertion, older adults are hypothesized to increasingly focus on preventing the loss of resources for less valuable goals. From this perspective, lower motivation for effortful behavior in older adults stems from changes in the goal structure in addition to the gradual loss of neural tissue. That is, instead of promoting the engagement in rewarded effort as in younger adults, the FPC might strengthen the goal to avoid costly expenditure of resources in older adults.

To determine the role of the FPC for motivation in older adults, the current study combines correlative analyses of structural brain changes with transcranial direct current stimulation (tDCS). tDCS is a non-invasive brain stimulation technique that facilitates the determination of the functional roles of a brain region by assessing how changing its excitability causally alters behavior. If the FPC preserves its computational role in motivating engagement in rewarded effort in older adults despite reduced GMV (as we originally hypothesized in line with the assumptions of aging neuroscience), increasing FPC excitability should enhance the willingness to engage in rewarded effort in older adults, similar to previous findings in younger adults (Soutschek *et al.*, 2018). However, contrary to our original hypothesis and to our findings for younger adults, the current data provide evidence that increasing FPC excitability leads to a lower willingness to exert effort in older adults. This is further supported by exploratory analyses, suggesting an age-specific relationship between FPC GMV and willingness to exert effort. By combining correlative structural analyses with causal brain stimulation methods, our study joins neural with psychological accounts of decision-making in aging, substantiating the notion that changes in goal setting crucially influence altered decision-making in older adults in addition to cortical thinning (Samanez-Larkin *et al.*, 2007).

## Materials and methods

### Participants

#### Older adults

The sample of healthy older participants included a total of 26 volunteers (*M*_age_ = 69.2 years, s.d._age_ = 4.2, range = 65–80, 12 female). Participants were recruited via the University of the Third Age (UZH3) at the University of Zurich. According to an a priori power analysis, a sample size of 30 participants allows detecting a significant tDCS effect with a power of 80% (alpha = 5%, one-tailed), assuming the effect size (Cohen’s *d* = 0.46) for the main effect of anodal *vs* sham tDCS in our study on younger adults (Soutschek *et al.*, 2018). It is worth noting that effect sizes from single previous studies may overestimate the true effect size in the population. However, due to the outbreak of the coronavirus-19 (COVID-19) pandemic, we were able to collect the data of only 26 volunteers before the pre-registered study termination date in June 2020, which reduced the statistical power to 74%. Because also after the termination date the ongoing pandemic impeded assessing further older adults as members of a high-risk group for COVID-19, we decided to terminate the study and analyze the data. For study enrollment, volunteers had to be aged between 65 and 80 years, have a score ≥27 in the Montreal Cognitive Assessment (MoCA; Nasreddine *et al.*, 2005) test and no history of psychological or neurological disorders. We note that a cutoff score of 27 in the MoCA has routinely been used in past studies, although more recent evidence suggests that it might be relatively high (Elkana *et al.*, 2020). Participants furthermore had to fulfill all inclusion criteria for tDCS and imaging experiments. These criteria included no history of psychiatric or neurological disorders, stroke, heart attack, or head injuries, and no metal in the body or head. The study was approved by the Cantonal Ethics Committee of Zurich, and the design and the hypotheses of the tDCS study were pre-registered on clinicaltrials.gov (identifier: NCT03197181). We obtained written informed consent from all participants before participation. Participants were reimbursed with 30 Swiss francs per hour plus a performance-dependent monetary bonus (see below).

#### Younger adults

We compared effort preferences in the older adults group with two separate samples of younger adults. The first sample of younger adults (control group 1) performed the mental and physical effort tasks under FPC tDCS, and the results for this dataset have already been reported (Soutschek *et al.*, 2018). To compare tDCS effects between younger and older adults, we selected the younger adults in the anodal tDCS (*N* = 43, *M*_age_ = 22.5, s.d._age_ = 3.0, range = 18–30, 22 female) and sham tDCS (*N* = 48, *M*_age_ = 23.2, s.d._age_ = 2.3, range = 20–30, 24 female) groups but discarded the cathodal stimulation group because the older adults cohort comprised only anodal and sham tDCS conditions.

Because in the first sample of younger adults structural scans were available only for a minority of participants, we decided, post hoc, to collect data from a second control group of younger adults in order to explore whether differences in GMV explain age-related differences in effort preferences. Control group 2 included 28 younger adults (*M*_age_ = 24.1 years, s.d._age_ = 3.0, range = 19–33, 14 female). A power analysis based on a previous study on age differences in effort-based decision-making (Westbrook *et al.*, 2013) suggested that 23 participants per age group should be sufficient to detect a significant difference with a power of 80% (alpha = 5%, one-tailed), assuming a Cohen’s *d* of 0.76 observed for the age × effort-level interaction in this study. All younger adults in control group 2 performed the physical effort decision task (see below) and underwent magnetic resonance imaging (MRI) to collect a structural brain scan, but they did not receive tDCS during task performance. For study enrollment, participants in control group 2 had to be aged between 18 and 35 years and fulfill the inclusion criteria for MRI.

### Stimuli and task design

**Fig. 1.**
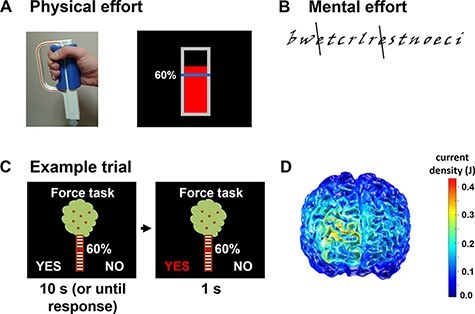
Experimental tasks. Older adult participants performed both a physical effort task and a mental effort task and younger adults performed the physical effort task only. (A) For physical effort exertion, participants had to squeeze a handgrip with variable levels of their maximum grip force for 20 s. (B) The mental effort exertion required crossing all letters ‘e’ according to a given rule in a text with a random letter sequence. Levels of mental effort were manipulated via the number of lines to be completed (with 20% defined as the number of lines a participant completed within 2 min). (C) In the effort-based decision task, participants made a series of choice between accepting and rejecting offers to engage in rewarded mental or physical effort. The magnitude of the available reward was illustrated by the number of apples on a tree, while required effort demands were indicated at the tree trunk. (D) Current density modeling suggests that tDCS effects were most pronounced in the right frontopolar cortex.

In the ‘effort-based decision task’ (Figure 1), participants decided whether or not they were willing to exert mental or physical effort for different monetary rewards (Soutschek *et al.*, 2018). For physical effort exertion (Figure 1A), participants had to squeeze a handgrip dynamometer for 20 s with 20%−100% of their individually determined maximum grip force (Soutschek *et al.*, 2018, 2020). For mental effort exertion (Figure 1B), participants had to cross all instances of the letter ‘e’ in a text composed of groups of random letters (i.e. pseudo-words) according to a demanding rule (the two letters before and the two letters after an ‘e’ must not comprise vowels). In analogy to calibrating physical effort levels to the individual maximum strength, the mental effort levels were calibrated to the individual performance level as follows: participants had to work on the text task for 2 min, and the number of lines completed within these 2 min was defined as 20% mental effort level. For example, if a participant completed 4 lines within 2 min, 20% mental effort in the decision task required the performance of 4 lines of this task, whereas 100% mental effort required 20 lines.

To make the task as intuitive as possible, we symbolized the monetary rewards that participants could obtain in a given trial by the number of red apples on a tree. We used five reward magnitudes (1, 3, 6, 9 and 12 apples) and informed participants that one apple would be exchanged for 0.1 Swiss francs after the experiment. The five possible effort levels were illustrated with white bars at the tree trunk (20%, 40%, 60%, 80% and 100% effort), and the effort level required in a given trial was indicated by a red bar at the corresponding height of the trunk (Figure 1C). Participants had to accept or reject the presented offer by pressing the left or right arrow key on a standard keyboard (key-choice assignment was counterbalanced across participants) within 10 s. The chosen option turned red for 1 s, and then, the next trial started. We administered separate blocks for mental and physical effort, with the current type of effort displayed on top of the tree.

To disentangle motivation to exert effort from exhaustion, participants did not exert the effort immediately after accepting an offer (Soutschek *et al.*, 2018, 2020; Westbrook *et al.*, 2020). Instead, one trial of the decision task was randomly selected at the end of the experiment and implemented. If participants had accepted the offer, they had to exert the corresponding amount of effort to obtain the monetary bonus. If they had rejected the offer, they did not have to exert effort after the experiment and received no bonus. We informed participants that each choice had an equal chance of being selected after the experiment. To avoid influences of risk aversion of effort-based choices (e.g. participants might fear to be unable to exert 100% physical effort for 20 s), participants could try to exert the required effort until they succeeded.

### Procedure

Participants from the older adult cohort took part in three experimental sessions: in the first session, we screened participants for exclusion criteria and collected a structural T1 scan. In sessions 2 and 3, participants performed the effort-based decision task while undergoing anodal or sham tDCS over the FPC (see below). At the start of these sessions, we familiarized participants with the required mental and physical work and calibrated task difficulty to individual performance levels. To remind them of the mental effort, they had to complete as many lines of text as possible during 2 min, whereas to illustrate physical effort, they had to squeeze the dynamometer for 20 s with 50% of their maximum grip force. Next, participants were instructed about the effort-based decision task, and they performed five trials for each effort type as practice.

During tDCS, participants performed four blocks of the mental and physical effort decision tasks (two blocks per effort type). Each block contained 25 trials, with all combinations of reward magnitudes and effort levels being presented once within a block. At the end of the experiment, participants rated their current mood and the perceived aversiveness of the stimulation, but these measures showed no significant differences between anodal and sham tDCS, mood: *t* < 1, *P* = 0.38, perceived aversiveness of stimulation: *t* < 1, *P* = 0.60.

The group of younger adults participated in one experimental session only where they performed the physical effort-based decision task (without tDCS). Younger adults performed only the physical effort-based decision task because this task allows for a more stringent calibration of effort levels to the individual maximum performance (i.e. maximum grip strength) than the mental effort task, which is a crucial precondition for comparing choice behavior across age groups.

### tDCS protocol

In two separate sessions, older adults received anodal or sham tDCS (in counterbalanced order) using a 16-channel tDCS stimulator (neuroConn, Ilmenau, Germany). A smaller, 5 × 5-cm electrode was placed over the right FPC via neuronavigation using the same Montreal Neurological Institute (MNI) coordinates (*x* = 32, *y* = 54, *z* = 21) as in our previous study (Soutschek *et al.*, 2018). A larger, 10 × 10-cm electrode was placed over the vertex, defined by the middle of the nasion–inion distance (see Figure 1D for electrode positioning and the modeled current density for anodal stimulation using the Comets2 toolbox; Jung *et al.*, 2013). We used a larger vertex than the FPC electrode to minimize the stimulation effect at the ‘control’ vertex site relative to the FPC site (Nitsche *et al.*, 2007). Both electrodes were fixed by rubber straps.

During the performance of the effort-based decision task, we stimulated with 1 mA current strength in the anodal tDCS condition, whereas for sham tDCS, we turned the current off after 30 s. Participants started task performance 4 min after the start of the stimulation to account for possible delays in the onset of tDCS effects.

### Magnetic resonance imaging

We collected high-resolution whole-brain T1-weighted images with a fast field echo sequence (number of slices = 170 slices, repetition time = 3.7 ms, echo time = 8 ms, voxel size = 1 × 1 × 1 mm^3^, flip angle = 8°, field of view = 256 × 256 mm^2^). These scans were acquired on a Philips Achieva 3T whole-body scanner with a 32-channel head coil (Philips Medical Systems, Best, The Netherlands) at the Laboratory of Social and Neural Systems Research (University of Zurich, Switzerland).

### Behavioral analyses

Behavioral data in the effort-based decision tasks were analyzed with mixed generalized linear models (MGLMs) using the lme4 package (Bates *et al.*, 2015) in R version 3.6.1. The alpha threshold was set to 5%. For all MGLMs, we used the optimizer ‘nloptwrap’ and increased the number of iterations to 2e^6^ in order to ensure model convergence. In addition, we computed Bayes factors as indicators of how strongly the data favor the alternative over the null hypothesis (BF_10_) with the brms package (function ‘hypothesis’). As prior for the null hypothesis, we assumed Cauchy-distributed priors centered around zero. To assess whether FPC tDCS modulates effort preferences in older adults, we regressed binary choices in the decision tasks on fixed-effect predictors for tDCS (0 = sham, 1 = anodal), Effort type (−1 = mental effort, 1 = physical effort), Effort level (*z*-standardized), Reward magnitude (*z*-standardized) and the interaction terms assessing how tDCS and Effort type modulate Effort level and Reward magnitude (MGLM-1). All main effects and interaction terms were also modeled as random slopes in addition to participant-specific random intercepts. Due to technical issues with the tDCS stimulator in the anodal session, both the anodal and sham data of two participants were excluded from this analysis.

We also compared the effects of tDCS on effort preferences between our current cohort of older adults and our previously published dataset involving younger adults (control group 1). To assess whether tDCS effects depend on age, we conducted an MGLM on the combined datasets (MGLM-2) that included the same predictors as MGLM-1 and additionally predictors for Age (*z*-standardized) as well as the interactions between Age and all predictors of MGLM-1.

We note that MGLM-2 does not allow comparing the influences of reward magnitude and required effort between younger and older adults due to differences in task procedures. We therefore compared effort preferences in the older adult cohort with a second control group of younger adults, which had performed the physical effort task in the same way as the older adults. To test the hypothesis that willingness to engage in effort decreases with increasing age, we regressed younger and older adults’ binary choices in the effort-based decision task on *z*-transformed fixed-effects predictors for Age, Effort level, Reward magnitude and all interaction terms (MGLM-3). As random effects, we modeled participant-specific random intercepts and random slopes for Effort level, Reward magnitude and the interaction term. In the cohort of older adults, we restricted this analysis to the data from the sham session. As post hoc tests, we computed MGLMs that assessed the impact of age on willingness to exert effort separately for each effort level (MGLM-4). For these post hoc tests, we adjusted the *P* values using Bonferroni correction (*P*_corrected_ = *P*_uncorrected_ × 5, which is equivalent to a corrected alpha threshold of ${{5\% } \over 5}$ = 1%).

### Voxel-based morphometry analysis

Preprocessing of the structural brain scans was performed using the CAT12 toolbox (Gaser and Dahnke, 2016) and comprised three steps. Each structural image first was normalized to MNI space and, second, segmented into gray matter (GM), white matter and cerebrospinal fluid. Third, the normalized segmented images were spatially smoothed with a Gaussian kernel (full width at half maximum = 8 mm). We also determined each participant’s total intracranial volume (TIV) using the routines implemented in the CAT12 toolbox.

To test whether differences in motivation between older and younger adults are related to changes in brain structure, we conducted a whole-brain voxel-based morphometry (VBM) analysis (using SPM12) regressing preprocessed GM voxels on the following predictors: acceptance rates for 20% effort (based on the individual coefficients from MGLM-3 for 20% effort), age and the interaction between age and acceptance rates. We used the 20% level as for this effort level we observed a significant age difference in motivation. As covariate of no interest, we entered the participant-specific TIV. In order to assess GMV changes in the FPC region stimulated in the tDCS experiment, we performed small-volume correction with a spherical region of interest (diameter = 16 mm, twice the smoothing kernel) centered at the coordinates used for the placement of the FPC tDCS electrode (*x* = 32, *y* = 54, *z* = 21). For exploratory whole-brain analyses, we controlled for multiple comparisons using family-wise error correction at the peak or cluster level, employing a cluster-inducing threshold of *P* < 0.001 (Eklund *et al.*, 2016).

## Results

### Anodal stimulation over FPC lowers willingness to engage in effort in older adults

**Fig. 2.**
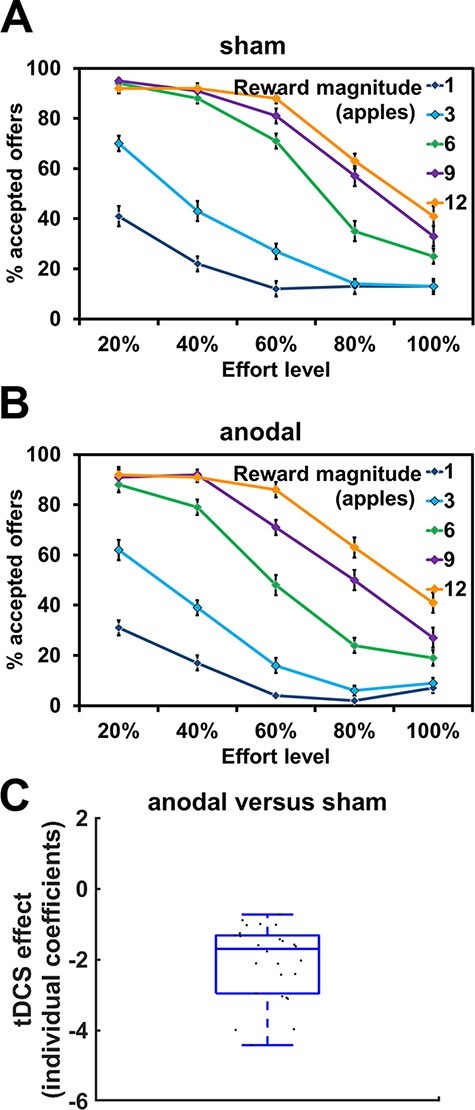
Brain stimulation results for older adults. (A, B) Illustration of the effects of frontopolar stimulation on willingness to exert effort as a function of effort level and reward magnitude, separately for (A) sham and (B) anodal stimulation (collapsed across mental and physical effort because tDCS effects did not significantly differ between mental and physical effort). Error bars indicate standard error of the mean. (C) Contrary to previous findings for younger adults (Soutschek *et al.*, 2018), anodal (relative to sham) tDCS over frontopolar cortex lowers the motivation to engage in rewarded mental or physical effort in older adults. Black dots indicate individual coefficients for the main effect of tDCS in MGLM-1.

We tested the computational role of the FPC for motivation by assessing whether excitatory anodal FPC tDCS increases the willingness to exert effort. MGLM-1 suggested that participants were less willing to engage in effort with increasing effort levels, beta = −2.94, *z* = 3.97, *P* < 0.001, BF_10_ = 2e28, as well as with decreasing rewards available, beta = 3.05, *z* = 4.21, *P* < 0.001, BF_10_ = 1e18, and effort discounting was steeper the higher the rewards at stake, beta = −0.89, *z* = 2.21, *P* = 0.04, BF_10_ = 52.6 (Figure 2A and B). Contrary to our findings for younger adults and our original hypothesis (Soutschek *et al.*, 2018), a main effect of tDCS indicated that older adults were less, rather than more, willing to exert effort under anodal compared with sham tDCS, beta = −1.99, *z* = 2.43, *P* = 0.02, BF_10_ = 3.9 (Figure 2C and Table 1). Note that if as prior for the Bayes factor we assumed the effect size from our previous study on younger adults (instead of a prior centered around zero), the Bayes factor of 4.4 would even more strongly favor the alternative over the null hypothesis (the likelihood of the null hypothesis, i.e. that the magnitude of the tDCS effect is equal to the tDCS effect in the younger adults study, relative to the alternative hypothesis is only 0.23). While there was a non-significant trend for higher acceptance rates for physical than for mental effort, beta = 0.61, *z* = 1.83, *P* = 0.07, BF_10_ = 4.3, tDCS effects did not significantly differ between mental and physical effort, beta = −0.26, *z* = 0.64, *P* = 0.52, BF_10_ = 2.1. No further effect including the factor tDCS was significant, all *z* < 0.39, all *P* > 0.39, and we also observed no significant differences between mental and physical effort (besides the trend-level main effect of Effort type), all *z* < 0.52, all *P* > 0.60. Taken together, increased FPC excitability lowered the motivation to engage in effort in older adults, in contrast to the role of the FPC in younger adults (Soutschek *et al.*, 2018).

**Table 1.** Results of MGLM-1 assessing the impact of frontopolar tDCS (anodal *vs* sham) on willingness to exert effort in older adults

	Beta (SE)	*z*	*P*	BF_10_
Intercept	1.62 (0.56)	2.89	0.004	
Reward	3.05 (0.72)	4.21	<0.001	1e18
Effort	−2.94 (0.74)	3.97	<0.001	2e28
Effort type	0.61 (0.33)	1.83	0.07	4.3
tDCS	−1.99 (0.89)	2.24	0.02	3.9
tDCS × reward	−0.60 (0.94)	0.64	0.52	2.2
tDCS × effort	0.94 (1.17)	0.81	0.42	1.3
tDCS × effort type	−0.26 (0.41)	0.64	0.52	2.1
Reward × effort	−0.89 (0.42)	2.11	0.04	52.6
Effort type × reward	0.04 (0.35)	0.13	0.90	0.5
Effort type × effort	−0.05 (0.43)	0.12	0.91	1.8
tDCS × effort type × reward	−0.11 (0.41)	0.27	0.79	1.2
tDCS × effort type × effort	0.06 (0.50)	0.12	0.90	3.6
tDCS × reward × effort	0.55 (0.64)	0.85	0.39	0.8
Effort type × reward × effort	−0.17 (0.34)	0.51	0.61	0.4
tDCS × effort type × reward × effort	0.19 (0.40)	0.47	0.64	0.5

BF_10_ is the Bayes factor indicating how strongly the data favor the alternative over the null hypothesis (note that no Bayes factor can be computed for fixed-effect intercepts in mixed models).

To directly assess whether FPC stimulation has opposite effects on effort-based decisions in younger *vs* older adults, we compared the current data in older adults with our previous tDCS dataset for younger adults (for details, see Soutschek *et al.* (2018)). The sample of younger adults included 43 participants in the anodal group and 48 participants in the sham group (we note that the sample of younger adults was considerably larger than the sample of older adults). The cathodal stimulation group was discarded from this analysis, because we had used only anodal and sham tDCS in the study on older adults. MGLM-2 on the combined data set revealed a significant interaction between Age and tDCS (anodal *vs* sham), beta = −0.28, *z* = 2.19, *P* = 0.03, BF_10_ = 3.8, providing further evidence that FPC tDCS has dissociable effects on effort-based decisions in younger and older adults. We also observed a significant age × effort-level interaction, beta = 0.63, *z* = 3.81, *P* < 0.001, BF_10_ = 96.4, suggesting that effort preferences under sham differed between younger and older adults as a function of the required effort level (Table 2). We note although that the tasks used in the older adults and the younger adults samples were not fully comparable, because we had used different calibration procedures for mental effort and because the task for younger adults had included additional reward and effort levels. As these differences might affect effort preferences, the comparison between effort preferences in older and younger adults based on these data should be interpreted with caution. Nevertheless, the findings provide preliminary and consistent evidence that the FPC might have dissociable roles for the motivation of effortful behavior in older and younger adults.

**Table 2.** Results of MGLM-2 comparing the impact of frontopolar tDCS (anodal *vs* sham) on willingness to exert effort between younger and older adults

	Beta (SE)	*z*	*P*	BF_10_
Intercept	−0.84 (0.28)	2.19	0.03	
Reward	3.73 (0.22)	16.61	<0.001	2.0e17
Effort	−3.68 (0.18)	−20.28	<0.001	4.4e16
Effort type	0.76 (0.12)	6.11	<0.001	3.4e17
tDCS	0.35 (0.26)	1.34	0.18	1.6
Age	0.48 (0.37)	1.30	0.19	3.7
tDCS × reward	0.16 (0.17)	0.95	0.34	1.4
tDCS × effort	0.08 (0.14)	0.55	0.59	0.9
tDCS × effort type	−0.06 (0.10)	0.61	0.54	0.7
tDCS × Age	−0.28 (0.13)	2.19	0.03	3.8
Reward × effort	−0.58 (0.10)	6.00	<0.001	781.3
Effort type × reward	0.14 (0.09)	1.61	0.11	0.6
Effort type × effort	0.19 (0.11)	1.66	0.10	1.8
Reward × age	−0.57 (0.21)	2.74	0.006	41.7
Effort × age	0.63 (0.16)	3.81	<0.001	96.4
Effort type × age	−0.17 (0.11)	1.58	0.11	10.3
tDCS × effort type × reward	0.07 (0.06)	1.10	0.27	0.9
tDCS × effort type × effort	−0.08 (0.09)	0.91	0.36	0.7
tDCS × reward × effort	−0.01 (0.08)	0.15	0.88	0.6
Effort type × reward × effort	−0.18 (0.07)	2.59	0.01	1.8
tDCS × reward × age	−0.03 (0.09)	0.33	0.74	0.5
tDCS × effort × age	−0.03 (0.07)	0.42	0.67	0.5
tDCS × effort type × age	0.03 (0.05)	0.55	0.58	0.4
Reward × effort × age	−0.01 (0.09)	0.16	0.88	0.5
Effort type × reward × age	−0.08 (0.06)	1.37	0.17	1.1
Effort type × effort × age	0.02 (0.09)	0.19	0.85	0.6
tDCS × effort type × reward × effort	−0.01 (0.06)	0.25	0.80	0.5
tDCS × effort type × reward × age	0.03 (0.04)	0.77	0.44	0.4
tDCS × effort type × effort × age	−0.05 (0.05)	1.02	0.31	0.9
tDCS × reward × effort × age	0.03 (0.04)	0.66	0.51	0.4
Effort type × reward × effort × age	0.08 (0.05)	1.38	0.17	0.4
tDCS × effort type × reward × effort × age	−0.00 (0.03)	0.02	0.98	0.3

BF_10_ is the Bayes factor indicating how strongly the data favor the alternative over the null hypothesis (note that no Bayes factor can be computed for fixed-effect intercepts in mixed models).

### Changes in the FPC structure explain individual differences in cost–benefit weighting in an age-specific manner

The finding that increased FPC excitability in older adults reduced motivation raises the question as to why FPC stimulation shows opposite effects on motivation in older *vs* younger adults. Given the hypothesized role of the FPC for integrating costs and benefits for action alternatives (Mansouri *et al.*, 2017), one possibility is that this result is due to a shift in goals from gain maximization to effort minimization, consistent with psychological theories of aging (Depping and Freund, 2011; Hennecke and Freund, 2017). The FPC activity may serve to enhance or boost the current goal over its costs, instead of simply promoting reward over effort in a fixed, goal-independent manner. This sort of goal-dependent role could explain why the FPC activity would result in reward-seeking overriding effort costs more often in younger adults (resulting in a higher willingness to engage in effort), whereas in older adults, it seems to promote effort avoidance.

To test for goal-dependent influences of the FPC in another way, we explored the relation between FPC GMV and behavior in older *vs* younger adults. Studies linking structural brain changes to age differences in decision-making or cognition typically assume that a region’s computational role remains constant over age but gradually declines (West, 1996; Li *et al.*, 2007; Dennis and Cabeza, 2012; Samanez-Larkin and Knutson, 2015; Lighthall, 2020). Contrary to this assumption, our tDCS findings suggest that correlations between the FPC structure and willingness to exert effort over age may not only reflect age-related decreases in functional efficacy in performing cost–benefit computations but also reflect a shift from gain to loss orientation. Together, the theories that goals switch from gain maximization to loss minimization (Depping and Freund, 2011; Hennecke and Freund, 2017) and that the FPC is goal-dependent predict that the relationship between FPC GMV and willingness to exert effort changes with age. Specifically, in younger adults, FPC GMV should be associated with a higher willingness to exert effort, whereas in older adults, higher FPC GMV should correlate with a lower willingness to engage in effort.

**Fig. 3.**
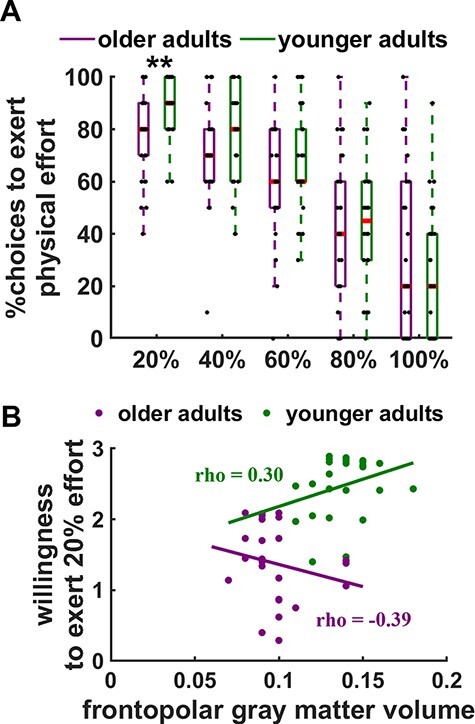
Relation of willingness to exert physical effort, age and frontopolar cortex volume. (A) Boxplots showing the willingness to exert physical effort as function of effort level (20%–100%), separately for younger and older adults. Older adults show a significantly lower willingness to exert low levels (i.e. 20% of maximum effort) of physical effort for rewards than younger adults. Red lines indicate the median, and black dots represent individual data points. (B) The relationship between the willingness to exert low levels of effort and frontopolar (FPC) gray matter volume (GMV) differed between younger and older adults. For display purposes, we show the younger and older adults as separate groups in different colors. While in younger adults higher FPC GMV tends to predict increased willingness to exert effort (green dots and regression line), in older adults greater FPC GMV is associated with a lower motivation to engage in rewarded effort (purple dots and regression line). Asterisks indicate significant effects (*P < 0.05, **P < 0.01).

To test this assumption, we collected data from a new group of younger adults, who—just as our cohort of older adults did—performed a physical effort-based decision task and underwent MRI for a structural brain scan. We first aimed to replicate previous findings that older adults are more effort-averse than younger adults (Hess and Ennis, 2012; Westbrook *et al.*, 2013) by regressing binary choices to accept *vs* reject effortful offers in the physical effort-based decision task on continuous predictors for Age, Effort level, Reward magnitude and the interaction terms (MGLM-3; Table 3). In the sample of older adults, we restricted this analysis to the physical effort-based task in the sham condition, because control group 2 of younger adults had performed only the decision task for physical effort without receiving tDCS (but undergoing MRI to collect individual structural scans; see the ‘Materials and methods’ section). A significant age × effort-level interaction, beta = 0.69, *z* = 2.01, *P* = 0.04, BF_10_ = 5.5, suggested that younger and older adults differed in their motivation to engage in effort depending on the required demands (Figure 3A). Post hoc MGLMs (MGLM-4; separately for each effort level) revealed that older adults had reduced acceptance rates for the lowest, 20% effort level, beta = −0.57, *z* = 3.38, *P* = 0.004, Bonferroni-corrected, BF_10_ = 57.3. The 40% effort level showed a non-significant trend for higher acceptance rates in the younger than in the older adult cohort, beta = −0.37, *z* = 2.39, *P* = 0.07, Bonferroni-corrected, BF_10_ = 2.9. For all other effort levels, we observed no significant age differences, all *z* < 1.37, all *P* > 0.8, Bonferroni-corrected. There was no evidence that age modulated the sensitivity to monetary rewards, beta = −0.29, *z* = 0.89, *P* = 0.37 BF_10_ = 1.3. Taken together, these findings suggest that aging is associated with increased sensitivity to low levels of effort, a hallmark of apathy (Le Heron *et al.*, 2018b).

**Table 3.** Results of MGLM-3 assessing the impact of age on willingness to exert physical effort

	Beta (SE)	*z*	*P*	BF_10_
Intercept	1.82 (0.34)	5.39	<0.001	
Reward	3.51 (0.36)	9.78	<0.001	2e17
Effort	−3.39 (0.38)	8.95	<0.001	4e20
Age	−0.36 (0.32)	1.14	0.25	1.6
Age × effort	0.69 (0.34)	2.01	0.04	5.5
Age × reward	−0.29 (0.32)	0.89	0.37	1.3
Reward × effort	−0.79 (0.25)	3.13	0.002	23.8
Age × reward × effort	−0.12 (0.24)	0.49	0.62	1.1

BF_10_ is the Bayes factor indicating how strongly the data favor the alternative over the null hypothesis (note that no Bayes factor can be computed for fixed-effect intercepts in mixed models).

Next, we asked whether these age-related differences in behavior can be explained by variation in brain structure. To test whether the link between FPC GMV and motivation changes with age, we regressed FPC GMV on predictors for age, willingness to exert effort (given by the individual coefficients from the MGLM for the 20% effort level, as this model had shown significant age differences in behavior) and the interaction term (controlling for TIV). We observed a significant interaction effect between age and willingness to exert effort in the FPC (*x* = 24, *y* = 62, *z* = 27; *t* = 3.87; *P* = 0.03, small-volume corrected with 16-mm spherical mask centered at coordinates for the FPC tDCS electrode placement; note that an exploratory whole-brain analysis revealed no further significant clusters, see Table 4). Plotting this interaction effect by extracting individual GMV from the significant FPC cluster suggests that in younger adults, as expected based on our previous findings, greater FPC GMV tended to predict higher willingness to exert effort, Spearman’s rho = 0.30, *P* = 0.06, one-tailed, BF_10_ = 1.9, whereas in older adults, FPC GMV was negatively correlated with acceptance rates, rho = −0.39, *P* = 0.046, two-tailed, BF_10_ = 5.8 (Figure 3B). Although these effects are relatively weak, they are nevertheless consistent with the observed opposite effects of FPC tDCS on motivation in younger and older adults.

**Table 4.** Anatomical locations and MNI coordinates of the peak activations showing an interaction effect between age and willingness to engage in 20% physical effort

			MNI coordinates				
**Region**	**Hem**	**BA**	***x***	***y***	***z***	***K***	**t**
Frontopolar cortex	R	10	24	62	27	20	3.87
Insula	R	13	36	−6	2	29	3.55
Supplementary motor area	L	6	−9	−14	63	21	3.55

The frontopolar cortex cluster is significant after applying small-volume correction within a spherical mask centered on the coordinates for the FPC tDCS electrode placement (see main text). The results in the insula and supplementary motor area do not survive corrections for multiple comparisons at the whole brain level (*P* < 0.001 uncorrected, minimum cluster size *k* ≥ 20 voxels) but are reported here for completeness and to facilitate the formation of future hypotheses and replication studies. We do not make any inferences on these insula and supplementary motor area results in the current manuscript. Hem = hemisphere (L = left, R = right); BA = Brodmann area.

## Discussion

Reduced motivation is a prevalent problem in aging, but its neurobiological roots remain unclear so far. Here, we provide preliminary evidence that changes in both brain structure and the goal orientations underlying cost–benefit trade-offs need to be considered in order to explain the lower willingness to engage in goal-directed effort in older adults. First, we replicated previous findings of increased effort aversion in older compared with younger adults (Hess and Ennis, 2012; Westbrook *et al.*, 2013), in line with increased apathy (Le Heron *et al.*, 2018b). We note that two previous studies reported no evidence for stronger physical effort aversion in older compared with younger adults (Seaman *et al.*, 2016, 2018), although this might partially be explained by a floor effect because effort aversion was rather low in all age groups in these studies. Interestingly, older adults showed stronger effort aversion than younger adults particularly to low effort requirements, which may potentially reflect a floor effect (as acceptances rates for high effort in younger adults were already rather low). Noteworthy, there was no evidence that the influence of reward magnitude on choices differed between age groups, speaking against the possibility that older adults valued monetary rewards less than younger adults. Many neuroscientific accounts of decision-making in older adults suggest that such behavioral deficits result from neural changes (Samanez-Larkin and Knutson, 2015; Lighthall, 2020), implicitly assuming that goals and neural computations remain constant throughout adulthood, and only few studies considered psychological accounts of aging to explain age-related changes in neural functioning (Samanez-Larkin *et al.*, 2007). Psychological theories posit that increased effort aversion in older adults reflects a switch from gain maximization to loss minimization goals (Depping and Freund, 2011; Hennecke and Freund, 2017).

Our study provides a further step toward bridging this gap by suggesting that changes in both brain structure and goal orientation contribute to the increased effort sensitivity in aging. First, increasing FPC excitability lowered the willingness to engage in mental or physical effort in older adults. This is in contrast to our own previous findings for younger adults, in which FPC stimulation increased motivation (Soutschek *et al.*, 2018). We note that previous studies on motor function, language processing and memory have reported similar effect sizes for stimulation effects in younger and older adults (or, if anything, even stronger stimulation effects in older adults) despite GMV loss in older adults (for a review, see Perceval *et al.*, 2016). Therefore, the opposite stimulation effects are unlikely to be caused by age-related changes in FPC GMV *per se*. Second, the tDCS findings are further supported by the VBM analysis providing evidence that the relationship between FPC GMV and willingness to exert effort changes with age, such that younger *vs* older adults show positive *vs* negative correlations between FPC GMV and motivation for rewarded effort (although we note that the correlations between GMV and motivation within each age group are relatively weak and should be interpreted with caution).

These findings are inconsistent with the assumption that the FPC plays a functional role in promoting reward obtainment that remains constant with age. Instead, a goal-dependent role for the FPC appears to be the best explanation for the age-dependent link between motivation and the FPC. We speculate that the FPC may bias the evaluation of reward benefits *vs* effort costs in different directions due to differences in internal goals in younger and older adults. This is consistent with psychological theories claiming that goals change from gain to loss orientation over the life span (Depping and Freund, 2011), although as caveat we note that we did not directly measure goal orientation in the current study. We also note that our study followed a cross-sectional instead of a longitudinal design, such that one needs to be careful with conclusions regarding changes in the FPC’s functional role on an individual level. Neurally, this shift in the FPC’s computational role may be linked age-related changes in the input and output between the FPC and brain regions representing rewards and effort costs. For example, aging appears to be associated with impairments in the dopaminergic reward system (Li and Rieckmann, 2014) and with increased functional connectivity strength between the FPC and supplementary motor cortex (Li *et al.*, 2020), a region that encodes physical effort costs and sends effort cost information to the FPC (Burke *et al.*, 2013; Bonnelle *et al.*, 2016). Thus, increased aversion to low levels of effort in older adults may also be a consequence of re-organization within motivation networks.

These conclusions have implications for neural accounts of decision-making across the life span. Such accounts often aim to relate quantitative properties of neural tissue (like GMV, cortical thickness or fiber connections) with the degree of cognitive functioning but neglect that goals, perhaps instantiated by a brain region’s relative weighting of inputs, may also change with increasing age (but see Samanez-Larkin *et al.*, 2007). We note that evidence for similar dissociations between younger and older adults was reported in other stimulation studies as well. For example, cathodal stimulation of the left and anodal stimulation of the right lateral prefrontal cortex reduces risk-taking in younger adults (Fecteau *et al.*, 2007), while older adults show a stronger preference for risky prospects under the same stimulation protocol (Boggio *et al.*, 2010). These findings too can be reconciled with the hypothesized change from gain maximization to loss avoidance over the life span. Thus, an implication of our results is that future neural investigations of decision-making in older adults should consider changes both in neural tissue and in goal orientation as mediators of altered behavior in aging.

It is worth mentioning some limitations of the current investigation. First, we emphasize the exploratory nature of the comparisons between younger and older adults, which we conducted to explain the unexpected tDCS effects in older adults. Second, while some of the *P* values for the tDCS effects and the group comparisons are relatively close to the statistical threshold, it is worth pointing out that the tDCS and the GMV findings provide converging evidence for age-specific roles of the FPC for motivation. Nevertheless, given the limited sample size and given that we could assess four older adults less than planned a priori due to the COVID-19 pandemic, both the tDCS and the GMV findings should be considered preliminary. Third, the mental effort task confounded effort with delay because higher effort levels required participants to work on more lines of text, which increased the delay until payment by a few minutes. However, the fact that we observed no significant differences between mental and physical effort suggests that time considerations had virtually no impact on participants’ choices.

Fourth, previous studies observed that the strength of tDCS effects depends on the cortical thickness of the stimulated region and/or on neurochemical concentrations of GABA or glutamate (Filmer *et al.*, 2019, 2020). Because in the current study we did not measure neurochemical concentrations and had acquired a structural scan only for a minority of the younger adults in the tDCS experiment, it is possible that differences in neurochemical concentrations or cortical thickness between older and younger adults could explain the dissociable effects of anodal tDCS in these groups. In fact, concentrations of neurotransmitters such as GABA or dopamine differ between younger and older adults (Mora *et al.*, 2008; Huang *et al.*, 2017) and might contribute to age-related changes in decision-making and reward sensitivity (Samanez-Larkin and Knutson, 2015). From this view, neurochemical changes constitute a plausible neurobiological mechanism underlying the FPC’s age-related change in functioning observed in our study.

A further limitation of the current findings is that the age difference in effort preferences was significant only for the lowest (20%) effort level. While we suggested that this result might hint a potential floor effect (e.g. 43% of all younger adults had an acceptance rate of 0% for the 100% effort level), there are other plausible explanations as well. According to the selective engagement theory (Hess, 2014), when deciding to mobilize effort, older adults, compared to younger adults, are more strongly guided by the personal relevance of goals or by self-presentation concerns. As it has been suggested that individuals occasionally engage in high effort demands for self-presentation purposes (Inzlicht *et al.*, 2018), a potential (albeit speculative) hypothesis for the lack of age differences at higher effort levels is that high effort demands more strongly activated self-presentation motives in older adults than in younger adults. In any case, we acknowledge that the current data do not provide a conclusive answer to the question as to why the age difference in effort avoidance was strongest for the lowest effort level.

Taken together, our findings provide insights into the psychological and neurobiological roots of altered motivation in healthy aging. One source of the increased avoidance of low effort in older adults might be a goal-dependent functioning of the FPC. Given the apparent goal-dependent nature of the FPC function and most likely other brain regions as well, neural interventions aimed at preventing or counteracting loss of motivation in older adults should consider how goals are set and pursued as well.

## Data Availability

The raw behavioral data, a script for the behavioral data analysis and the results of the VBM analysis are available on Open Science Framework (https://osf.io/ejs47/). The tDCS experiment was preregistered on clinicaltrials.gov (identifier: NCT03197181).
